# Risk Factors for Patient-Important Upper Gastrointestinal Bleeding

**DOI:** 10.1164/rccm.202411-2245OC

**Published:** 2025-04-18

**Authors:** Adam M. Deane, François Lauzier, Neill K. J. Adhikari, François Lamontagne, Diane Heels-Ansdell, Lehana Thabane, David Williamson, Salmaan Kanji, Jeffrey F. Barletta, Simon Finfer, Yaseen Arabi, Marlies Ostermann, John C. Marshall, Nicole L. Zytaruk, Miranda Hardie, Naomi E. Hammond, Gordon Guyatt, Kyle C. White, Karen E. A. Burns, Joanna C. Dionne, Paul J. Young, Deborah J. Cook

**Affiliations:** ^1^ Department of Critical Care, Melbourne Medical School, University of Melbourne, Melbourne, Victoria, Australia;; ^2^ Département de médecine et Département d’anesthésiologie et de soins intensifs, Université Laval, Québec, Québec, Canada;; ^3^ Interdepartmental Division of Critical Care Medicine, University of Toronto, Toronto, Ontario, Canada;; ^4^ Département de soins intensifs, Université de Sherbrooke, Sherbrooke, Québec, Canada;; ^5^ Department of Health Research Methods, Evidence and Impact, McMaster University, Hamilton, Ontario, Canada;; ^6^ The Research Institute of St Joseph’s Healthcare, Hamilton, Ontario, Canada;; ^7^ Département de Pharmacie, Hôpital du Sacré-Cœur, Université de Montréal, Montréal, Québec, Canada;; ^8^ The Ottawa Hospital Research Institute, Ottawa, Ontario, Canada;; ^9^ Department of Pharmacy Practice, Midwestern University, College of Pharmacy-Glendale Campus, Glendale, Arizona, USA;; ^10^ Critical Care Program, The George Institute for Global Health, University of New South Wales, Sydney, Australia;; ^11^ School of Public Health, Imperial College London, London, England;; ^12^ Intensive Care Department, Ministry of the National Guard - Health Affairs, King Saud bin Abdulaziz University for Health Sciences, King Abdullah International Medical Research Center, Riyadh, Kingdom of Saudi Arabia;; ^13^ Department of Intensive Care Medicine, Guys and St. Thomas’ Hospital, London, England;; ^14^ Malcolm Fisher Department of Intensive Care, Royal North Shore Hospital, St Leonards, Australia;; ^15^ Intensive Care Unit, Princess Alexandra Hospital, Woolloongabba, Australia;; ^16^ Medical Research Institute of New Zealand, Wellington, New Zealand; and; ^17^ Intensive Care Unit, Wellington Hospital, Wellington, New Zealand

**Keywords:** critical illness, gastrointestinal bleeding, risk, stress ulcer prophylaxis

## Abstract

**Rationale:**

Patient-important gastrointestinal bleeding is an endpoint developed by patients and family members; however, risk factors for this outcome are unknown.

**Objectives:**

We sought to identify risk factors for patient-important upper gastrointestinal bleeding among invasively ventilated adults.

**Methods:**

This preplanned regression analysis of an international trial database evaluated baseline and time-varying risk factors in the preceding 3 days for patient-important upper gastrointestinal bleeding, accounting for illness severity and the competing risk of death.

**Measurements and Main Results:**

Patient-important upper gastrointestinal bleeding occurred in the ICU among 131 of 4,821 (2.7%) patients. Baseline APACHE II score—hazard ratio (HR), 1.24 (95% confidence interval [CI] = 1.12, 1.37) per 5-point increase—and the following were associated with greater risk of patient-important bleeding: inotropes or vasopressors (HR, 2.05 [95% CI = 1.35, 3.12]), severe thrombocytopenia (platelet count, <50 × 10^9^/L) (HR, 2.21 [95% CI = 1.24, 3.94]) and platelet inhibitor drugs (HR, 1.69 [95% CI = 1.11, 2.56]). A lower bleeding risk was associated with pantoprazole (HR, 0.36 [95% CI = 0.25, 0.54]) and enteral nutrition (HR, 0.81 [95% CI = 0.68, 0.97]) for every increase of 500 ml/d. There was no interaction between enteral nutrition and pantoprazole (interaction *P* = 0.94). Allocation to pantoprazole was associated with a lower risk of patient-important upper gastrointestinal bleeding regardless of the volume of enteral nutrition (for 500 ml/d: HR, 0.36 [95% CI = 0.22, 0.58]; for no enteral nutrition: HR, 0.36 [95% CI = 0.18, 0.72]). The association of enteral nutrition and bleeding was similar with pantoprazole (HR, 0.82 [95% CI = 0.63, 1.07]) or without pantoprazole (HR, 0.81 [95% CI = 0.66, 1.00]).

**Conclusions:**

Several factors are associated with the risk of patient-important upper gastrointestinal bleeding during invasive ventilation.

**At a Glance Commentary**

**Scientific Knowledge on the Subject**

Patient characteristics that predispose to upper gastrointestinal bleeding are numerous and variably selected, analyzed, and reported in prior studies. Risk factors for upper gastrointestinal bleeding that is important to ICU survivors and family members, reflecting contemporary practice, are unclear.

**What This Study Adds to the Field**

In this large international database, an increased risk of patient-important upper gastrointestinal bleeding among invasively ventilated adults was associated with higher illness severity, inotropes or vasopressors, severe thrombocytopenia, and platelet aggregation inhibitors. Pantoprazole and increasing amounts of enteral nutrition were associated with a lower risk of patient-important upper gastrointestinal bleeding. Volume of enteral nutrition did not attenuate the effectiveness of pantoprazole at bleeding prevention.

Critically ill patients are at risk of stress erosions in the upper gastrointestinal tract that may cause clinically important upper gastrointestinal bleeding (1–5). Although many bleeding risk factors have been reported, the epidemiology and care of critically ill patients has changed over time, and no study has evaluated risk factors for bleeding using an endpoint defined by ICU survivors and family members. Most research evaluating gastrointestinal bleeding as a complication of critical illness has reported *clinically important* upper gastrointestinal bleeding as defined by study investigators (1–5). In response to the infrequent use of endpoints that are determined by members of the public in clinical research (6, 7), the outcome of *patient-important* upper gastrointestinal bleeding was developed in a mixed-methods study by survivors of critical illness and family members of patients admitted to an ICU (8).

Unlike clinically important upper gastrointestinal bleeding, hemodynamic changes and hemoglobin values associated with bleeding were not part of the definition. ICU survivors and relatives of patients who experienced critical illness defined patient-important upper gastrointestinal bleeding as overt upper gastrointestinal bleeding that required a single blood transfusion, vasopressor treatment, diagnostic endoscopy, computed tomography angiography, or surgery, or that resulted in death, disability, or prolonged hospitalization (9).

Patient-important upper gastrointestinal bleeding was a secondary endpoint for a recent international stress ulcer prophylaxis trial (10). REVISE (Re-Evaluating the Inhibition of Stress Erosions) was a multicenter, randomized, stratified, concealed, parallel-group trial that enrolled 4,821 invasively ventilated patients who were at least 18 years old and admitted to the ICU, designed to determine the effect of pantoprazole versus placebo on the primary efficacy outcome of clinically important upper gastrointestinal bleeding and the primary safety outcome of 90-day mortality (11, 12). In the main analysis, pantoprazole significantly reduced the risk of clinically important upper gastrointestinal bleeding and patient-important upper gastrointestinal bleeding (the numbers needed to prevent a bleeding episode were 40 and 37, respectively); other outcomes related to microbiome modification and the gastropulmonary route of infection (13) were unaffected (10).

Stress ulcer prevention in practice may range from no prophylaxism, to a risk-based approach, to universal prescribing (14, 15). The primary objective of this study was to identify, among invasively ventilated critically ill adults, the contemporary risk factors for patient-important upper gastrointestinal bleeding, considering illness severity, and the competing risk of death.

## Methods

### Ethics Approval

REVISE is approved by Health Canada (HC6-24-c210404), Clinical Trials Ontario Research Ethics Project ID: 1360, the Northern Sydney Local Health District Human Research Ethics Committee (HREC 2019/ETH08405), and Comissão Nacional de Ética em Pesquisa (CONEP 5.734.590). Regulatory oversight is by Health Canada (HC6-24-c210404). All participating centers have local ethics approval.

### Design

This preplanned secondary analysis of the REVISE trial (ClinicalTrials.gov ID: NCT 03374800) was guided by a publicly available protocol and statistical analysis plan (16) supported by the Canadian Critical Care Trials Group and the Australian and New Zealand Intensive Care Society Clinical Trials Group. This study was funded by peer-reviewed grants from the Canadian Institutes of Health Research, Accelerating Clinical Trials Canada, and National Health and Medical Research Council of Australia and the National Institute for Health and Care Network.

REVISE enrolled invasively mechanically ventilated patients who were expected to remain ventilated beyond the calendar day after randomization. Patients were excluded who *1*) had been invasively ventilated for more than 72 hours, *2*) received acid suppression for more than one daily dose equivalent in the ICU, *3*) received dual antiplatelet therapy or combined antiplatelet agent and therapeutic anticoagulation, *4*) actively or recently experienced upper gastrointestinal bleeding, or *5*) had another clear indication or contraindication to pantoprazole (10). Patients received blinded study drug (40 mg daily intravenous pantoprazole or placebo) while they were invasively ventilated unless a contraindication or clear indication developed for up to 90 days in the ICU. Eligible patients were enrolled by either *a priori* informed consent, deferred consent, or opt-out, aligned with local approvals. All management decisions were at the discretion of the treating ICU team. No additional ethical approval was needed for this preplanned analysis.

**Figure 1.**
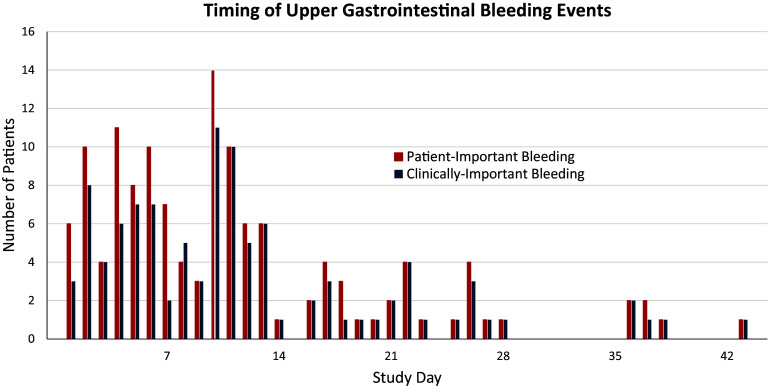
Timing of upper gastrointestinal bleeding events. Study day on which patient-important upper gastrointestinal bleeding and clinically important upper gastrointestinal bleeding occurred.

Daily data for the time-dependent risk factors were not collected beyond the index ICU admission, so the 5 patients who bled on the ward, prompting ICU readmission, were censored at ICU discharge. Therefore, this analysis focused on bleeds during the index ICU admission among 131 patients who had patient-important upper gastrointestinal bleeding and 104 patients who had clinically important upper gastrointestinal bleeding. The day of each upper gastrointestinal bleed is shown in Figure 1.

### Risk Factors

We included baseline variables and time-dependent variables, considering a suitable number of independent variables to avoid an over-fitted model (17). All baseline variables were obtained before patients received the study drug. All time-dependent risk factors were considered in the preceding 3 days but did not include the day of bleed. For bleeding that occurred on Day 1 of daily data collection, we used only Day 1 data for time-dependent covariates (16).

Baseline variables included *1*) the Acute Physiology and Chronic Health Evaluation (APACHE) II score; *2*) a medical versus surgical or trauma admission diagnostic category; and *3*) pantoprazole versus placebo as the randomized group.

Time-dependent variables measured during the ICU stay included *1*) respiratory failure, defined as receiving invasive mechanical ventilation; *2*) circulatory failure, defined as receiving inotropes or vasopressors (any dose of any of norepinephrine, epinephrine, phenylephrine, vasopressin, dopamine, dobutamine or milrinone); *3*) renal failure, defined as end-stage renal disease or acute kidney injury newly receiving renal replacement therapy (any of intermittent hemodialysis, continuous renal replacement therapy, sustained low efficiency dialysis or peritoneal dialysis); *4*) daily enteral nutrition, defined as volume administered in milliliters per day; *5*) therapeutic anticoagulation, defined as treatment doses of unfractionated heparin or low– molecular weight heparin, fondaparinux, warfarin, non–vitamin K antagonist oral anticoagulants (including anti-Xa inhibitors and direct thrombin inhibitors, targeting systemic anticoagulation), and thrombolytic therapy; *6*) coagulopathy, defined as international normalized ratio higher than 3.0 or prothrombin time more than 70 seconds; *7*) severe thrombocytopenia, defined as a platelet count less than 50 × 10^9^/L; *8*) platelet aggregation inhibitors, defined as receipt of any of acetylsalicylic acid, clopidogrel, dipyridamole, ticlopidine, tirofiban, P2Y12 inhibitors, or nonsteroidal anti-inflammatory drugs at any dose; and *9*) corticosteroids, defined as any corticosteroid drug, regardless of dose, administered enterally or intravenously. These potential risk factors were selected on the basis of evidence review and investigator discussion.

*A priori*, we postulated that the following would be associated with an increased risk of bleeding: higher APACHE II score, circulatory failure, renal failure, therapeutic anticoagulation, coagulopathy, severe thrombocytopenia, platelet aggregation inhibitors, and corticosteroids. We postulated that the variables receipt of pantoprazole and enteral nutrition would be associated with a decreased risk of bleeding. As invasive ventilation was an inclusion criterion for REVISE, and extubation is typically rapidly followed by ICU discharge, an insufficient number of days without ventilatory support would render risk factor analysis of invasive ventilation underpowered; thus, we postulated that invasive mechanical ventilation would not be associated with bleeding in this cohort. We did not include the following baseline characteristics in the main model to avoid overfitting, and postulating no clear association with bleeding: acute hepatic failure (after adjusting for severe thrombocytopenia and coagulopathy), severe traumatic brain injury, and sex.

### Outcomes

The primary outcome of patient-important upper gastrointestinal bleeding in the ICU has been defined previously in a mixed-methods study of ICU survivors and families who considered bleeding to be important if it required a single blood transfusion, vasopressor treatment, diagnostic endoscopy, computed tomography angiography, or surgery or if it resulted in death, disability, or prolonged hospitalization (8, 9).

Also, to analyze the conventional bleeding outcome in this field, we included clinically important upper gastrointestinal bleeding in the ICU as a secondary outcome. This was defined as overt bleeding (hematemesis, overt nasogastric bleeding, melena, or hematochezia) and at least one of the following within 24 hours in the absence of another cause: spontaneous decrease in invasively monitored mean arterial pressure or noninvasive systolic or diastolic blood pressure of 20 mm Hg or more or an orthostatic increase in pulse rate of 20 beats per minute and a decrease in systolic blood pressure of 10 mm Hg, with or without vasopressor initiation or increase; vasopressor initiation; a decrease in hemoglobin of ⩾20 g/L in a 24-hour period or less; transfusion of ⩾2 U of packed red blood cells within 24 hours of bleeding, or therapeutic intervention (e.g., angiography, surgery, or endoscopic treatment) (10).

**Figure 2.**
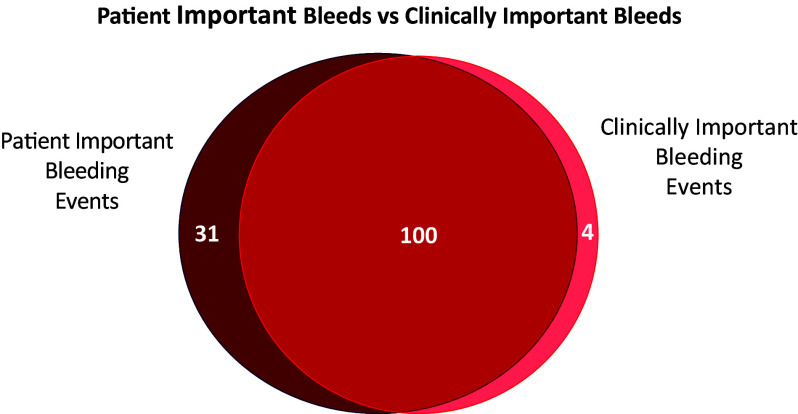
Patient-important bleeds versus clinically important bleeds. Relationship between upper gastrointestinal bleeding events that were patient important and those that were clinically important. Most bleeding events fulfilled both definitions. However, four events were adjudicated as clinically important bleeding on the basis of a decrease in hemoglobin ⩾20 g/L that did not fulfill criteria for patient-important bleeding. There were 31 patients with patient-important bleeding on the basis of criteria such as transfusion of 1 U packed red blood cells, or diagnostic but not therapeutic endoscopy, which did not qualify for clinically important bleeding.

In REVISE, all bleeding events reported by participating centers were reviewed in duplicate by an adjudication committee member who was blinded to the study drug to determine whether criteria for patient-important or clinically important upper gastrointestinal bleeding criteria were met (18). A summary of the definitional differences between patient-important and clinically important bleeding events is presented elsewhere (*see* Table E1 in the online supplement). The resultant relationship between patient-important and clinically important gastrointestinal bleeding events is shown in Figure 2.

### Statistical Analysis

To assess the effect of risk factors on the primary outcome of time to patient-important upper gastrointestinal bleeding, a Fine and Gray competing risk analysis was used (17, 19–21), accounting for death as a competing risk (22), with discharge from the ICU and consent withdrawal considered as censoring events.

There were few missing data except after 2 weeks on study, as platelet aggregation inhibitors and corticosteroids were not collected after Day 14. If a platelet aggregation inhibitor was received on Day 14, we imputed that it was continued until ICU discharge for the original indication, except in the event of bleeding. If a patient received corticosteroid treatment on Day 14, we imputed that the indication continued to Day 21 (16).

Regression results are reported as hazard ratios (HRs) and 95% confidence intervals (CIs) and two-tailed *P* values were calculated; *P* < 0.05 indicates statistical significance. No multiplicity corrections were performed (16). Analyses were performed using SAS Version 9.4.

### Sensitivity and Secondary Analyses

In sensitivity analyses, we conducted a Cox proportional hazards regression analysis to evaluate risk factors for patient-important upper gastrointestinal bleeding that did not consider the competing risk of death. Second, we evaluated all time-dependent risk factors within the preceding 2 days rather than 3 days, for exposures more proximate to the patient-important upper gastrointestinal bleed.

In secondary analyses, we evaluated risk factors for the secondary outcome of clinically important upper gastrointestinal bleeding using the same factors as in the main model. Separately, to evaluate whether enteral nutrition through enhanced mucosal integrity (23), attenuated or augmented the effect of pantoprazole on prevention of patient-important bleeding compared with placebo, we included an interaction term between pantoprazole versus placebo and amount of enteral nutrition received (24).

Finally, to evaluate whether certain baseline conditions were associated with patient-important upper gastrointestinal bleeding, we incorporated the following into the main model one at a time: acute hepatic failure (25), severe traumatic brain injury (26), and female sex (27).

## Results

Among 4,821 patients enrolled in 68 international ICUs, 131 patients developed patient-important upper gastrointestinal bleeding in the ICU (2.7%) and 104 patients developed clinically important upper gastrointestinal bleeding in the ICU (2.2%). The prevalence of patient characteristics and incidence of exposure related to potential bleeding risk factors are described in Table 1, reported among those with patient-important upper gastrointestinal bleeding and clinically important upper gastrointestinal bleeding.

**Table 1.** Incidence of Potential Risk Factors for Upper Gastrointestinal Bleeding

Variable	Patients without Patient-Important Upper GI Bleeding (*n* = 4,690)	Patients with Patient-Important Upper GI Bleeding (*n* = 131)	Patients without Clinically Important Upper GI Bleeding (*n* = 4,717)	Patients with Clinically Important Upper GI Bleeding (*n* = 104)
Prevalence of baseline variables				
APACHE II score, mean (SD)	21.6 (8.2)	25.9 (8.5)	21.6 (8.2)	26.5 (8.4)
APACHE II score ⩾25, *n* (%)	1,555 (33.2)	71 (54.2)	1,563 (33.1)	63 (60.6)
Admission type, *n* (%)				
Medical	3,420 (72.9)	100 (76.3)	3,437 (72.9)	83 (79.8)
Surgical	598 (12.8)	22 (16.8)	604 (12.8)	16 (15.4)
Trauma	672 (14.3)	9 (6.9)	676 (14.3)	5 (4.8)
Randomized group, *n* (%)				
Pantoprazole	2,383 (50.8)	34 (26.0)	2,394 (50.8)	23 (22.1)
Placebo	2,307 (49.2)	97 (74.0)	2,323 (49.2)	81 (77.9)
Incidence of time-dependent variables (ever while on study)				
Respiratory failure receiving invasive mechanical ventilation, *n* (%)	4,690 (100.0)	131 (100.0)	4,717 (100.0)	104 (100.0)
Circulatory failure receiving inotropes or vasopressors, *n* (%)	3,663 (78.1)	128 (97.7)	3,687 (78.2)	104 (100.0)
Renal failure receiving dialysis, *n* (%)	560 (11.9)	60 (45.8)	567 (12.0)	53 (51.0)
Enteral and/or oral nutrition in ml/d, median (IQR)	960 (500–1,428)	840 (300–1,320)	955 (500–1,425)	840 (300–1,330)
Therapeutic anticoagulation, *n* (%)	1,077 (23.0)	60 (45.8)	1,093 (23.2)	44 (42.3)
Coagulopathy, *n* (%)	474 (10.1)	47 (35.9)	487 (10.3)	34 (32.7)
Severe thrombocytopenia, *n* (%)	345 (7.4)	39 (29.8)	354 (7.5)	30 (28.8)
Platelet aggregation inhibitors, *n* (%)	970 (20.7)	37 (28.2)	978 (20.7)	29 (27.9)
Corticosteroids, *n* (%)	2,308 (49.2)	89 (67.9)	2,325 (49.3)	72 (69.2)
Additional baseline variables (for secondary analyses), *n* (%)				
Female	1,707 (36.4)	46 (35.1)	1,714 (36.3)	39 (37.5)
Admitting diagnosis of traumatic brain injury	394 (8.4)	5 (3.8)	396 (8.4)	3 (2.9)
Admitting diagnosis of acute hepatic failure	15 (0.3)	1 (0.8)	15 (0.3)	1 (1.0)

*Definition of abbreviations*: APACHE II score = Acute Physiology and Chronic Health Evaluation II score (a severity of illness score ranging from 0 to 71; higher scores indicate an increased risk of death); IQR = interquartile range.

Table 1 shows the prevalence of potential bleeding risk factors considered in the analysis, shown among 131 participants with a patient-important upper gastrointestinal bleed and 104 participants with a clinically important upper gastrointestinal bleed. For definitions, please refer to the main text.

The proportional hazards assumption was assessed and met (19). No collinearity of the independent variables was found.

Illness severity, as measured by the APACHE II score (for each 5-point increment: HR, 1.24 [95% CI = 1.12, 1.37]; *P* < 0.001), receipt of inotropes or vasopressors (HR, 2.05 [95% CI = 1.35, 3.12]; *P* < 0.001), severe thrombocytopenia (HR, 2.21 [95% CI = 1.24, 3.94]; *P* = 0.008), and platelet aggregation inhibitors (HR, 1.69 [95% CI = 1.11, 2.56]; *P* = 0.015) were independently associated with developing patient-important upper gastrointestinal bleeding. The association with antiplatelet agents reflects dominantly acetylsalicylic acid which was received on 13.6% of ICU-study days (dual antiplatelet agents were received for 1.4% of ICU-study days, which prompted discontinuing study drug and prescribing open-label proton-pump inhibitors) (Table 2).

**Table 2.** Risk Factors for Upper Gastrointestinal Bleeding in Invasively Ventilated Patients

Variable	Primary Outcome: Patient-Important Upper Gastrointestinal Bleeding		Secondary Outcome: Clinically Important Upper Gastrointestinal Bleeding	
	Hazard Ratio (95% CI)	*P* Value	Hazard Ratio (95% CI)	*P* Value
Baseline variables				
APACHE II score, per 5-U increase	1.24 (1.12, 1.37)	<0.001	1.26 (1.12, 1.41)	<0.001
Medical vs. surgical–trauma	0.87 (0.58, 1.31)	0.496	1.01 (0.61, 1.65)	0.979
Pantoprazole vs. placebo	0.36 (0.25, 0.54)	<0.001	0.30 (0.19, 0.48)	<0.001
Time-dependent variables				
Respiratory failure receiving invasive mechanical ventilation	1.20 (0.59, 2.46)	0.616	1.12 (0.52, 2.41)	0.778
Circulatory failure receiving inotropes or vasopressors	2.05 (1.35, 3.12)	<0.001	2.00 (1.25, 3.19)	0.004
Renal failure receiving dialysis	1.45 (0.93, 2.28)	0.102	1.73 (1.06, 2.83)	0.027
Enteral nutrition, per 500-ml increase	0.81 (0.68, 0.97)	0.022	0.82 (0.68, 0.98)	0.028
Therapeutic anticoagulation	1.31 (0.85, 2.03)	0.216	0.98 (0.59, 1.64)	0.938
Coagulopathy	1.52 (0.84, 2.75)	0.165	1.94 (1.01, 3.74)	0.046
Severe thrombocytopenia	2.21 (1.24, 3.94)	0.008	1.53 (0.76, 3.10)	0.234
Platelet aggregation inhibitors	1.69 (1.11, 2.56)	0.015	1.57 (0.98, 2.51)	0.060
Corticosteroids	1.24 (0.85, 1.81)	0.269	1.51 (0.97, 2.33)	0.068

*Definition of abbreviations*: APACHE II score = Acute Physiology and Chronic Health Evaluation II score (a severity of illness score ranging from 0 to 71; higher scores indicate an increased risk of death); CI = confidence interval.

Table 2 shows the risk factors for the primary outcome of patient-important upper gastrointestinal bleeding considering the competing risk of death. The right side of the table shows the risk factors for the secondary outcome of clinically important upper gastrointestinal bleeding considering the competing risk of death, as per the primary analysis. For definitions, please refer to the main text.

Stress ulcer prophylaxis with pantoprazole (HR, 0.36 [95% CI = 0.25, 0.54]; *P* < 0.001) and enteral nutrition during the ICU stay (HR, 0.81 [95% CI = 0.68, 0.97] for every 500-ml increase, *P* = 0.022, were associated with a lower risk of patient-important upper gastrointestinal bleeding (Table 2).

### Sensitivity Analyses

Conditions influencing the risk of patient-important upper gastrointestinal bleeding were the same when the competing risk of death was not considered (Table 3).

**Table 3.** Sensitivity Analyses: Risk Factors for Patient-Important Upper Gastrointestinal Bleeding

Variable	Risk Factors Not Considering the Competing Risk of Death		Risk Factors in the Preceding 2 instead of 3 Days	
	Hazard Ratio (95% CI)	*P* Value	Hazard Ratio (95% CI)	*P* Value
Baseline variables				
APACHE II score, per 5-U increase	1.25 (1.13, 1.39)	<0.001	1.25 (1.13, 1.38)	<0.001
Medical vs. surgical–trauma	0.90 (0.59, 1.35)	0.598	0.87 (0.58, 1.31)	0.502
Pantoprazole vs. placebo	0.37 (0.25, 0.55)	<0.001	0.36 (0.25, 0.54)	<0.001
Time-dependent variables	(in the preceding 3 d)		(in the preceding 2 d)	
Respiratory failure receiving invasive mechanical ventilation	1.26 (0.61, 2.58)	0.530	1.25 (0.67, 2.32)	0.479
Circulatory failure receiving inotropes or vasopressors	2.13 (1.39, 3.26)	<0.001	1.66 (1.11, 2.50)	0.015
Renal failure receiving dialysis	1.35 (0.88, 2.08)	0.167	1.57 (1.003, 2.47)	0.048
Enteral nutrition, per 500-ml increase	0.76 (0.64, 0.89)	<0.001	0.82 (0.68, 0.97)	0.022
Therapeutic anticoagulation	1.26 (0.82, 1.94)	0.298	1.26 (0.80, 1.97)	0.322
Coagulopathy	1.60 (0.92, 2.79)	0.095	1.83 (1.004, 3.33)	0.049
Severe thrombocytopenia	2.45 (1.40, 4.27)	0.002	2.24 (1.21, 4.12)	0.010
Platelet aggregation inhibitors	1.69 (1.12, 2.55)	0.013	1.64 (1.08, 2.51)	0.022
Corticosteroids	1.23 (0.84, 1.80)	0.298	1.23 (0.84, 1.81)	0.278

*Definition of abbreviations*: APACHE II score = Acute Physiology and Chronic Health Evaluation II score (a severity of illness score ranging from 0 to 71; higher scores indicate an increased risk of death); CI = confidence interval.

Table 3 shows the results of two sensitivity analyses examining risk factors for patient-important upper gastrointestinal bleeding. The first set does not consider the competing risk of death. The second set considers time-dependent risk factors in the preceding 2 days rather than 3 days. For definitions, please refer to the main text.

When considering time-dependent risk factors for bleeding within the preceding 2 days, results were similar to those of the primary analysis that considered the preceding 3 days, and two additional risk factors were identified: renal failure receiving dialysis (HR, 1.57 [95% CI = 1.00, 2.47]; *P* = 0.048) and coagulopathy (HR, 1.83 [95% CI = 1.00, 3.33]; *P* = 0.049) (Table 3).

### Secondary Analyses

Some conditions associated with clinically important upper gastrointestinal bleeding were concordant with those predisposing to patient-important bleeding, including illness severity, and circulatory failure receiving inotropes or vasopressors, pantoprazole, and enteral nutrition. Although severe thrombocytopenia and platelet aggregation inhibitors were not associated with an increased risk of clinically important upper gastrointestinal bleeding, additional risk factors were renal failure receiving dialysis (HR, 1.73 [95% CI = 1.06, 2.83]; *P* = 0.027) and coagulopathy (HR, 1.94 [95% CI = 1.01, 3.74]; *P* = 0.046).

There was no significant interaction between enteral nutrition and pantoprazole (interaction *P*  = 0.94). The risk of patient-important upper gastrointestinal bleeding was consistently lower in patients receiving pantoprazole, regardless of the amount of enteral nutrition (for 500 ml/d: HR, 0.36; 95% CI = 0.22, 0.58; for no enteral nutrition, HR, 0.36; 95% CI = 0.18, 0.72). The association of enteral nutrition and bleeding was similar whether pantoprazole was received (HR, 0.82; 95% CI = 0.63, 1.07) or not (HR, 0.81; 95% CI = 0.66, 1.00) (Table 4).

**Table 4.** Secondary Analyses: Pantoprazole and Enteral Nutrition

Variable	Patient-Important Upper Gastrointestinal Bleeding	
	Hazard Ratio (95% CI)	*P* Value
Baseline variables		
APACHE II score, per 5-U increase	1.24 (1.12, 1.37)	<0.001
Medical vs. surgical–trauma	0.87 (0.58, 1.31)	0.496
Time-dependent variables		
Respiratory failure receiving invasive mechanical ventilation	1.20 (0.59, 2.46)	0.617
Circulatory failure receiving inotropes or vasopressors	2.05 (1.35, 3.12)	<0.001
Renal failure receiving dialysis	1.45 (0.93, 2.27)	0.102
Therapeutic anticoagulation	1.31 (0.85, 2.03)	0.165
Coagulopathy	1.52 (0.84, 2.75)	0.165
Severe thrombocytopenia	2.21 (1.24, 3.94)	0.008
Platelet aggregation inhibitors	1.69 (1.11, 2.56)	0.015
Corticosteroids	1.24 (0.85, 1.81)	0.269
Interaction between pantoprazole vs placebo and the amount of enteral nutrition received per day	—	Interaction *P* = 0.944
Pantoprazole vs. placebo when 500 ml enteral nutrition is received	0.36 (0.22, 0.58)	—
Pantoprazole vs. placebo when no enteral nutrition is received	0.36 (0.18, 0.72)	—
Enteral nutrition (per 500-ml increase) in the pantoprazole group	0.82 (0.63, 1.07)	—
Enteral nutrition (per 500-ml increase) in the placebo group	0.81 (0.66, 1.00)	—

*Definition of abbreviations*: APACHE II = Acute Physiology and Chronic Health Evaluation II score (a severity of illness score ranging from 0 to 71; higher scores indicate an increased risk of death); CI = confidence interval.

Table 4 shows the risk factors for patient-important bleeding, including an interaction term between pantoprazole versus placebo and the amount of enteral nutrition received. For definitions, please refer to the main text.

When added to the main model including both coagulopathy and severe thrombocytopenia, acute hepatic failure (HR, 5.44 [95% CI = 0.76, 38.92]; *P* = 0.09), severe traumatic brain injury (HR, 0.62 (95% CI = 0.25, 1.54); *P* = 0.30), and female sex (HR, 0.96 (95% CI = 0.67, 1.39); *P* = 0.84) were not associated with patient-important upper gastrointestinal bleeding.

## Discussion

Several conditions predisposing invasively ventilated critically ill adults to patient-important upper gastrointestinal bleeding were identified in this study, including illness severity, treatment with inotropes or vasopressors, severe thrombocytopenia, and platelet aggregation inhibitors. We did not document a significantly increased risk of bleeding associated with corticosteroid treatment, congruent with mixed existing evidence (28–30).

Although both pantoprazole and increasing amounts of enteral nutrition were associated with a lower risk of patient-important upper gastrointestinal bleeding, volume of enteral nutrition did not attenuate the relative effect of pantoprazole for bleeding prevention. By contrast, gastrointestinal bleeding is rarely an outcome in large nutrition trials (31–34). Given the design of our study, the observed association of enteral nutrition with lower risk of patient-important upper gastrointestinal bleeding does not indicate causality or suggest discontinuing proton-pump inhibitors when enteral nutrition is established. Indication bias may be operant, and the association may also be related to unmeasured confounders such as gastrointestinal dysfunction.

In this study, sensitivity analyses considering risk factors in the previous 2 days instead of 3 days identified the same risk factors as the primary analysis, with the addition of renal failure receiving dialysis and coagulopathy as predisposing to patient-important upper gastrointestinal bleeding. Two other multicenter randomized trial databases have been used to analyze risk factors, both of which found that the bleeding diathesis inherent in renal dysfunction was a risk factor, but in these studies, for clinically important rather than patient-important upper gastrointestinal bleeding (30, 35). With a focus only on baseline characteristics among 3,291 patients in the SUP-ICU trial (4), logistic regression found that illness severity, measured using the Severe Acute Physiology Score II (36) and the Sequential Organ Failure Assessment score (37); use of circulatory support; and renal replacement therapy were associated with increased risk of bleeding (30). Evaluating both baseline characteristics and time-dependent factors in a 1,200-patient trial (2), univariable analyses suggested that thrombocytopenia and maximum renal, hepatic, and pulmonary domains and overall Multiple Organ Dysfunction score (38) predisposed to bleeding; however, multivariable analyses identified only maximum serum creatinine as an independent risk factor (35).

Building on risk factor evidence from prior trials and many other study designs (39), in this study, we use time-to-event modeling to assess events and exposures in the ICU while adjusting for illness severity and the competing risk of death to examine patient-important bleeding. Challenges interpreting studies of risk factors for clinically important upper gastrointestinal bleeding relate to different populations enrolled (influencing the profile and prevalence of each risk factor), risk factors themselves (various putative risk factors considered, alternate or unclear definitions), bleeding endpoints (different outcomes, different definitions, or ascertainment methods of the same outcome), and analytic methods (e.g., univariable analyses, overfitted multivariable models with low outcome event rates). It is also challenging in the current and prior studies to know whether risk factors predispose patients to bleeding, perpetuate the duration of bleeding, potentiate the severity of bleeding, or a combination thereof.

Although gastrointestinal bleeding in the ICU has an attributable mortality (40) and can be fatal, in most cases it is recognized early, is treated, and does not result in death. In the REVISE trial, pantoprazole did not decrease the risk of death (HR, 0.94 [95% CI = 0.85, 1.04]) (10), aligned with a meta-analysis of 12 trials (relative risk, 0.99 [95% CI = 0.93, 1.05]) (41). Subgroup analysis of this meta-analysis raised the possibility that pantoprazole may be associated with a higher risk of death among patients with greater illness severity compared those with lower illness severity (41) (effect modification between low and moderate credibility (42)). By contrast, the effectiveness of pantoprazole at reducing bleeding is evidenced, regardless of illness severity. The main analysis incorporated the competing risk of death.

Other strengths of this study include the first risk factor assessment of an outcome developed by critically ill patients and relatives of ICU survivors or decedents (9, 43). A novel feature of this endpoint is engagement of ICU survivors and family members to reflect on the tests and treatments for, as well as consequences of, upper gastrointestinal bleeding that matter to them. Through their lived experience, patients and families provide unique perspectives that may differ from and complement clinician perspectives. Scientifically and philosophically, learning from patients and family members, and integrating their views into studies, is a key step toward democratizing the research process. This study helps to fill a void in the critical care literature by selecting a primary outcome that is relevant to, and was developed by, patients and families situated in the broader context of their engagement in health care, quality improvement, research, and health policy.

The analysis did not deviate from the protocol (16). Beyond baseline characteristics, we evaluated exposures over the ICU stay using Cox regression while controlling for the competing risk of death and illness severity. This facilitated an organ and system severity-based approach; for example, renal failure requiring dialysis incorporated both pre-ICU end-stage renal disease and new acute kidney injury over time. Defining a time-dependent variable of circulatory failure receiving inotropes or vasopressors avoided the problem of overfitted regression models caused by evaluation of many unique admitting diagnoses (e.g., hypovolemic, septic, cardiogenic, obstructive, or anaphylactic shock) that prompt ICU admission or develop during the ICU stay. We selected variables for adjustment on clinical grounds, not on the basis of analytic output or statistical procedures. This placebo-controlled trial database allowed risk factor evaluation with and without stress ulcer prophylaxis. We also examined clinically important upper gastrointestinal bleeding and explored some proposed research questions on this topic (44). Heterogeneous participants enrolled in 68 international centers increase the generalizability and currency of these findings.

Limitations of this study include minimal information on some uncommon conditions such as exposure to only parenteral nutrition or unassisted breathing. This inception cohort was invasively ventilated; thus, it was not possible to assess bleeding risk associated with noninvasive ventilation which was used for only 2% of total ICU days. Given that only 16 patients with acute hepatic failure were randomized in this placebo-controlled trial, it is not surprising that this diagnosis was not independently associated with patient-important bleeding after adjusting for coagulopathy and severe thrombocytopenia (45) from any cause. Patients at extremely high risk of bleeding may not have been enrolled in this placebo-controlled randomized stress ulcer prophylaxis trial, thereby lowering the overall bleeding rate of the cohort, and possibly modifying the strength of association for risk factors identified. Also, we could not explore dose–response relationships of drug exposures in this analysis. This study was not designed to construct or validate a prediction model. The protocol and statistical analysis plan was publicly available on ClinicalTrials.gov as of September 18, 2024, and submitted for peer review on September 11, 2024, but was not peer reviewed when we started this analysis. Regression analyses may yield inflated or attenuated risk estimates due to confounding and are subject to residual biases, including confounder bias and collider bias. These results provide information on association rather than causation, warranting cautious interpretation.

### Conclusions

Several conditions are associated with an increased risk of patient-important upper gastrointestinal bleeding among invasively ventilated adults, including illness severity, treatment with inotropes or vasopressors, severe thrombocytopenia, and platelet aggregation inhibitors. Pantoprazole and increasing amounts of enteral nutrition were associated with a lower risk of patient-important upper gastrointestinal bleeding. The volume of enteral nutrition did not attenuate the relative effect of pantoprazole bleeding prevention. These findings may inform clinical decision making, practice guidelines, and future study design.

## Supplemental Materials

10.1164/rccm.202411-2245OC Online Data Supplement
