# Supplementary material for: Risk Factors for Patient-Important Upper Gastrointestinal Bleeding
Source: Am J Respir Crit Care Med. 2025 Apr 18;211(9):1671–80. doi: 10.1164/rccm.202411-2245OC (PMC12432427; doi:10.1164/rccm.202411-2245OC)
Supplement: Online Data Supplement [file rccm.202411-2245OCS1.docx]

**Risk Factors for Patient-Important Upper Gastrointestinal Bleeding**

Adam M Deane, MD, PhD, François Lauzier, MD, MSc, Neill KJ Adhikari, MDCM, MSc, François Lamontagne, MD, MSc, Diane Heels-Ansdell, MSc, Lehana Thabane, PhD, David Williamson, PhD, Salmaan Kanji, Pharm D, Jeffrey F Barletta, PharmD, Simon Finfer, MBBS, Yaseen Arabi, MD, Marlies Ostermann, MD, PhD, John C Marshall, MD, Nicole L Zytaruk, RN, Miranda Hardie, RN, MSc, Naomi E Hammond, RN, PhD, Gordon Guyatt, MD, MSc, Kyle C White, MBBS, MPH, Karen EA Burns, MD, Joanna C Dionne, MD, PhD, Paul J Young MBChB, PhD, Deborah J Cook, MD, MSc, for the Canadian Critical Care Trials Group and the Australian and New Zealand

**ONLINE DATA SUPPLEMENT**

**Supplemental Table 1:**

**Comparison of Clinically-Important and Patient-Important Gastrointestinal Bleeding**

| **Clinically-Important Gastrointestinal Bleeding** | **Patient-Important Gastrointestinal Bleeding** |
| --- | --- |
| **↓** SBP or ↓ DBP or ↓ MAP> 20mm/Hg with/without vasopressor initiation/increase | --- |
| Orthostatic ↓SBP > 10mm/Hg + ↑HR > 20/min with/without vasopressor initiation/increase | --- |
| Vasopressor initiation | Vasopressor initiation |
| ↓ Hemoglobin > 2g/dl | --- |
| Transfusion > 2 U RBCs | Transfusion > 1 U RBC |
| Therapeutic endoscopy | Endoscopy (diagnostic or therapeutic) |
| CT angio-embolization | CT angiography  (with/without embolization) |
| Surgery | Surgery |
| --- | Resulting in death |
| --- | Resulting in disability |
| Readmission to ICU | Resulting in prolonged hospitalization |

**Legend:**

This table shows similarities and differences between clinically-important upper gastrointestinal bleeding defined as per the REVISE Trial, and the primary outcome of the current study which is patient-important upper gastrointestinal bleeding.  These criteria are applicable in the presence of overt gastrointestinal bleeding and at least one of the criteria listed above within 24 hours of the overt bleeding, in the absence of other causes.
